# Glial Cells and Brain Diseases: Inflammasomes as Relevant Pathological Entities

**DOI:** 10.3389/fncel.2022.929529

**Published:** 2022-06-16

**Authors:** Esperanza Mata-Martínez, Mauricio Díaz-Muñoz, Francisco G. Vázquez-Cuevas

**Affiliations:** Laboratorio de Fisiología Celular, Departamento de Neurobiología Celular y Molecular, Instituto de Neurobiología, Universidad Nacional Autónoma de México, Juriquilla, Mexico

**Keywords:** inflammasome, glia, astrocyte, microglia, oligodendrocyte, NLRP3, neuroinflammation

## Abstract

Inflammation mediated by the innate immune system is a physiopathological response to diverse detrimental circumstances such as microbe infections or tissular damage. The molecular events that underlie this response involve the assembly of multiprotein complexes known as inflammasomes. These assemblages are essentially formed by a stressor-sensing protein, an adapter protein and a non-apoptotic caspase (1 or 11). The coordinated aggregation of these components mediates the processing and release of pro-inflammatory interleukins (IL-β and IL-18) and cellular death by pyroptosis induction. The inflammatory response is essential for the defense of the organism; for example, it triggers tissue repair and the destruction of pathogen microbe infections. However, when inflammation is activated chronically, it promotes diverse pathologies in the lung, liver, brain and other organs. The nervous system is one of the main tissues where the inflammatory process has been characterized, and its implications in health and disease are starting to be understood. Thus, the regulation of inflammasomes in specific cellular types of the central nervous system needs to be thoroughly understood to innovate treatments for diverse pathologies. In this review, the presence and participation of inflammasomes in pathological conditions in different types of glial cells will be discussed.

## Introduction

In response to pathogens or damage, the innate immune system of multicellular organisms responds with an alarm signal known as inflammation. Detection of stressor agent hints, like pathogen-associated molecular patterns (PAMPs, e.g., lipopolysaccharide (LPS), flagellin or viral RNA) or damage-associated molecular patterns (DAMPs, e.g., ATP, cytochrome C, defensins, galectins or uric acid), triggers the assembly of cytosolic multimeric protein complexes named inflammasomes. Inflammasomes process and release IL-β and IL-18 and induce pyroptotic cell death, a kind of sophisticated apoptosis directly related to inflammation (Bryant and Fitzgerald, 2009).

### Classification

**FIGURE 1:**
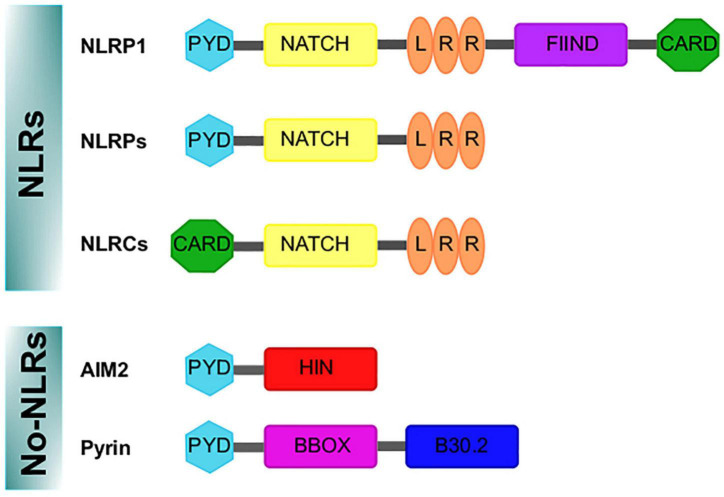
Domains structure of the inflammasomes sensor proteins. Nod like receptor (NLRs) proteins have a nucleotide-binding and oligomerization domain (NACHT/NBD), a leucine-rich repeat (LRR) motifs, typically located in the center and carboxy terminus of the NLR proteins, respectively. The NACHT motif is usually flanked by an additional amino-terminal domain, either a caspase recruitment domain (CARD) or a pyrin domain (PYD). For NLRP1, a FIIND and CARD domain are in the C-terminal position after the LRR domain. In addition, the absent in melanoma 2 (AIM2)-like receptor and Pyrin are no-NLR inflammasome sensors. AIM2 is characterized by an amino-terminal PYD domain and one or two DNA-binding HIN domains. Pyrin, features a PYD domain, two B-boxes, and a C-terminal B30.2 domain.

First described around 20 years ago (Martiñon et al., 2002), inflammasomes constitute a complex sharing functional and structural characteristics and are appointed and classified in function of the sensor protein triggering their assembly. Thus, inflammasomes can belong to those containing nucleotide-binding oligomerization domains (NOD/NATCH) and leucine-rich domains (NLRs), or those not containing the mentioned domains (no-NLRs) (Angosto-Bazarra et al., 2021; Zhang et al., 2021; Figure 1).

The NLR family includes NOD proteins, NOD-like receptor proteins (NLRPs) and NOD-like receptor C4 (NLRC4). Advances in NLR classification and nomenclature are available elsewhere (Mackenzie et al., 2008; Ting et al., 2008; Schroder and Tschopp, 2010). The NLR group is formed by 23 members in humans and 34 in mice; these proteins show characteristic motifs such as pyrin domain (PYD), a conserved central NATCH domain responsible for protein oligomerization, a carboxy terminal leucine-rich repeat (LRR) domain and a variable amino terminal region (reviewed in Walsh et al., 2014a) (Figure 1). NLRP1, NLRP2, NLRP3, NLRP4, NLRP6 and NLRP12 can participate in the formation of inflammasomes, being NLRP3 the best characterized (Rathinam et al., 2012). The importance of NLRP3 as a regulator of immune homeostasis is highlighted by the fact that mutations in the *NLRP3* gene cause autoimmune diseases such as the Muckle–Wells syndrome (also known as UDA syndrome) and the familial cold autoinflammatory syndrome (urticaria episodes triggered by exposure to cold) (Hoffman et al., 2001), collectively named cryopyrin-associated periodic syndromes (Broderick et al., 2015). Another relevant sensor protein is NLRC4 (originally named IPAF because it is related to APAF-1), which is characterized by a caspase recruitment domain (CARD), with high affinity for caspase-1, bound to NATCH and LRR domains (Figure 1); caspase-1 association promotes the autocatalytic activation of their protease activity (Poyet et al., 2001). NLRC4 is relevant in the context of microbial infection, as it can sense flagellin of *Salmonella typhimurium* (Amer et al., 2006) and is activated by a mechanism other than NLRP3, but in some circumstances both can be cooperative (Qu et al., 2016).

In addition, the absent in melanoma 2 (AIM2)-like receptors (AIM2) are other well-characterized no-NLR inflammasome sensors expressed in the central nervous system (CNS) (Heinisch et al., 2021). AIM2 protein is an inflammasome component specialized in the detection of mislocated or foreign DNA (from viruses, bacteria or other parasites). It has been demonstrated that disruption of the nuclear envelope also induces its aggregation (Di Micco et al., 2016); when the AIM2 inflammasome is assembled, it promotes pyroptosis (Lugrin and Martiñon, 2018).

### Structure

Inflammasomes are multiprotein complexes (∼700 kDa) constituted by three basic elements: (1) the previously described *cytosolic pattern recognition receptors*, (2) an *adapter protein* and (3) *an effector*, typically pro-caspase-1. For inflammasome aggregation, extracellular (i.e., ATP as a signaling molecule) or intracellular (i.e., uric acid, an adenosine catabolite) DAMPs trigger the activity of NLR; then NLR recruits the adapter protein apoptosis-associated speck-like protein containing a caspase recruitment domain (ASC), which contains PYD and CARD domains, to interact with the CARD domain of pro-caspase-1 (Bryant and Fitzgerald, 2009). The three components of the inflammasome unit can be self-assembled into filamentous structures with complex stoichiometries (Broderick et al., 2015).

**FIGURE 2:**
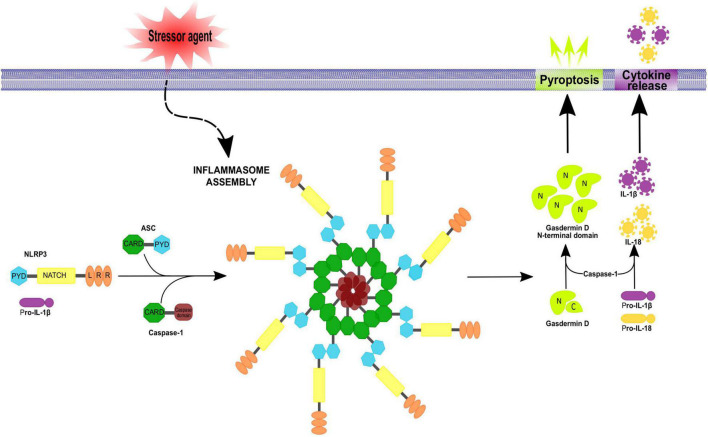
Inflammasome assembly. Inflammasomes assemble in a stimulus-specific manner. Different stressor agents are able to induce inflammasome activation by NLRs or no-NLR inflammasome sensors proteins. Activation of the NLRP3 inflammasome involves ASC and procaspase-1 recruitment, resulting in ASC oligomerization into a macromolecular aggregate and subsequent activation of procaspase-1. Active caspase-1 then cleaves pro-IL-1β and pro-IL-18 to their mature forms IL-1 β and IL-18 which get secreted. In addition, caspase-1 can cleave gasdermin D, releasing its N-terminal fragment which translocate to the plasma membrane inducing pore formation and pyroptotic cell death. The apoptosis-associated speck-like protein containing a caspase recruitment domain (ASC) is an adaptor protein for many inflammasome complexes and is composed of CARD and PYD domains, the latter being necessary for homotypic interaction with a PYD-containing inflammasome sensor. Procaspase-1 features a CARD domain, in addition to its caspase domain, and homotypic CARD interactions result in direct or indirect (*via* ASC) recruitment of procaspase-1 to the inflammasome complex.

Caspases are cysteinyl-aspartate proteases essential for inflammation, apoptosis and pyroptosis. In humans, the caspase family has 11 members. Caspases involved in inflammation are caspases 1, 4 and 5 in humans and 1, 11 and 12 in rodents (reviewed in Lamkanfi et al., 2007); recruitment of pro-caspase-1 induces its proteolytic processing to generate mature IL-β and IL-18 (Angosto-Bazarra et al., 2021; Zhang et al., 2021). Active caspase-1 also hydrolyzes Gasdermin D (GsdmD), the main protein related to pyroptosis execution, generating a fragment known as GsdmD N-terminal (Burdette et al., 2021), which forms pores in the plasma membrane that allow the entry of water toward the cytoplasm followed by an osmotic shock and cell outbreak, promoting the release of the cell content to the extracellular space including accumulated interleukins. In this context, caspase-1 activates other executer caspases to complete the death process by destroying nuclear DNA and cytoskeleton proteins (Bergsbaken et al., 2009; Figure 2).

### Activation

Activation of the inflammatory process by DAMPs is a well-understood event when extracellular ATP (eATP) is the trigger (Adinolfi et al., 2018). When this nucleotide is accumulated in the extracellular space, it binds to the P2X7 receptor activating the depolarizing influx of Ca^2+^ and Na^+^ as well as the efflux of K^+^; the ATP-induced reduction in cytoplasmic K^+^ leads to NLRP3 activation *via* NEK7 kinase, promoting NLRP3 inflammasome assembly (Pétrilli et al., 2007; He et al., 2016). P2X7 receptor activation also induces the formation of a pore with high conductance that allows the transit of molecules of ∼1 kDa; the stabilization of this *megapore* involves the participation of pannexin-1 (Pelegrin and Surprenant, 2006). Since pannexin-1 has been detected in association with the NLRP2/P2X7 receptor complex (Minkiewicz et al., 2013), it could be a player of P2X7 receptor-dependent inflammasome activation. Moreover, the increment in reactive oxygen species (ROS) by P2X7 receptor also favors inflammasome activation (Hung et al., 2013). In addition, protein-protein interaction between P2X7 receptor and the scaffold protein NLRP3 has been demonstrated (Franceschini et al., 2015), suggesting a cooperative mechanism between different cellular events elicited by P2X7 receptor to accomplish inflammasome activation.

It has also been described that the inflammasome activated by LPS mediates ATP/P2X7 receptor-dependent pyroptosis; thus, LPS administration induces caspase-11 activation, which cleaves pannexin-1 originally located in the plasma membrane. This proteolytic event induces a dramatic increment in the eATP triggering NLRP3 inflammasome activation, which guides the cell to pyroptosis. In agreement with the role played by the multiprotein complex of P2X7 receptor-dependent inflammasomes, LPS priming was unable to induce pyroptosis in *Panx1^–/–^, P2X7^–/–^* or *Casp11^–/–^* mice (Yang et al., 2015).

Acute activation of inflammatory responses helps the injured tissue to restore homeostasis or overcome pathogen infections; however, ample evidence supports the notion that chronic inflammation is a harmful mechanism that contributes to the development of various nervous system pathologies such as Alzheimer’s disease (AD; Heneka et al., 2013), stroke (Fann et al., 2013), depression (Iwata et al., 2013), autism (Saresella et al., 2016a), bipolar disorder (Kim et al., 2015a), sclerosis (Keane et al., 2018) and Parkinson’s disease (PD; Wang et al., 2022). Expression and regulation of inflammasomes in glial cells in this pathological context will be discussed.

## Inflammasomes in Microglial Cells

Microglial cells are considered specialized resident macrophages of the brain, showing particular genetic expression patterns compared with circulating macrophages (Lavin et al., 2014) and self-renewal within the brain (Ajami et al., 2007). Microglial cells have diverse functions, such as clearing apoptotic cells by phagocytosis and supporting synaptogenesis by secreting neural factors during the learning process (Labzin et al., 2018).

In their resting state, microglial cells display a basal stellated morphology and establish contact with neurons, astrocytes and endothelial cells; but when an injury or a metabolic challenge occurs, they change their morphology to acquire an amoeboid shape with high migratory abilities to reach the damaged zone and secrete pro-inflammatory mediators that promote astrocytic activation and recruitment of peripheral immune cells (Colonna and Butovsky, 2017). Microglia can secrete proinflammatory cytokines like IL-1β, IL-6, tumor necrosis factor alpha (TNF-α), IL-23, and IL-18 in response to DAMPs, including PAMPs, endogenous alarmins, and misfolded proteins (Prinz et al., 2019). Moreover, they are equipped with a set of receptors, called the microglial sensome, which allow the recognition of invading pathogens, misfolded proteins, chemokines and cytokines, metabolites, inorganic substances, and changes in pH or extracellular matrix components (Hickman et al., 2013). The murine microglial sensome comprises purinergic receptors (P2X4, P2X7, P2Y12, P2Y13, and P2Y6), chemokine receptors (CCR5, CX3CR1, CXCR4, and CXCR2), Fc receptors (FCERer1G and FCGR3), interferon-induced transmembrane proteins (IFITM2, IFITM3, and IFITM6), Toll-like receptors (TLR2 and TLR7), and Siglecs (SiglecH and Siglec3/CD33) (Prinz et al., 2019).

Regarding inflammasome machinery, it has been reported that LPC-dependent inflammation specifically induces caspase-1 activity mediated by NLRP3 and NLRP4 inflammasomes in microglia (Scholz and Eder, 2017). Additionally, the expression of NLRP3 components (*NLRP3, Asc* and *casp-1*) was detected by polymerase chain reaction in primary cultures of microglial cells, suggesting that inflammasomes are functional in these cells. On another hand, treatment of microglial cells with DAMPs, such as ATP, nigericin or alum, induces IL-1β production, supporting the role played by microglial cells in inflammasome aggregation (Gustin et al., 2015).

Furthermore, cooperative actions of AIM2 and NLRP3 have been observed when microglial cells were primed by ligands derived from *Brucella abortus*; AIM2 senses *Brucella* DNA and NLRP3 inflammasomes detect ROS (Marim et al., 2017) contributing to an immunopathological state and generating a disease named neurobrucellosis.

Taken together, these observations indicate that modulation of the immune response by microglia is critical to maintaining homeostasis in the brain.

### Inflammasome Activation in Microglial Cells

Aggregation and activation of inflammasomes is a general mechanism to face different types of cell injury; within the brain, it has been suggested that the immune response begins in microglial cells. A wide variety of stimuli that promote the activation of inflammasomes involve many pathogens and different classes of DAMPs.

When microglial cells face a pathogen, for instance *Streptococcus pneumoniae*, a microorganism whose infections produce meningitis, they respond by assembling inflammasome NLRP3, activating caspase-1, secreting IL-1β and IL-18 and inducing cell death by pyroptosis. In addition, the infection is associated with autophagy; nevertheless, the relationship with pyroptosis is not well understood (Kim et al., 2015b).

Likewise, when infection by HIV affects the CNS, a sustained NLRP3 inflammasome-dependent response leads to neurological damage specifically affecting microglial cells. By using microglia-derived cell lines, researchers observed that HIV promotes the synthesis of pro-IL-1β after 4 h post-infection and the release of this cytokine after 24 h; in agreement, brains of patients with AIDS showed high levels of IL-1β, IL-18 and caspase-1 (Walsh et al., 2014b). These data have been replicated in a model of feline immunodeficiency virus infection of cats, showing that IL-1β induction and microglial activation are associated with the occurrence of neurobehavioral deficits. Further, it was found that the HIV-1 transactivator of transcription (Tat) protein is instrumental for the upregulation of NLRP3 and ASC levels themselves and for the increase in caspase-1 cleavage and subsequent IL-1β release (Chivero et al., 2017). Similarly, the HIV-1 viral protein R (Vpr) can induce NLRP3-dependent caspase-1 cleavage and IL-1β release. Furthermore, treatment of Vpr transgenic animals with the caspase-1 inhibitor VX-765 improved neurobehavioral deficits (Mamik et al., 2017).

On the other hand, prions are misfolded endogenous proteins whose accumulation induces serious neurological disorders. One of the most common prion diseases in bovines and goats is *scrapie*, characterized by uncontrolled movements, altered behavior and finally death. The prionic disease is mediated by inflammation, unleashed in response to the fragment of the protein PrP*^sc^* (PrP*^sc^* 106-126). It involves the activation of the NLPR3 inflammasome in microglial cells primed with LPS, producing the release of IL-1β (Shi et al., 2012). Additionally, stimulation of microglia with PrP fibrils was also shown to induce toxicity in neurons (Hafner-Bratkovič et al., 2012). Mechanistically, NLRP3 inflammasome activation was suggested to negatively regulate autophagy in microglia, thereby contributing to neurodegeneration (Lai et al., 2018). However, controversial results in mice have shown that the activity of NLRP3 inflammasome is not necessary for establishing *scrapie*; thus, the effect of intraventricular injection of PrP*^sc^* 106-126 was identical in knockout mice for *NLRP3* and *pycard* (the gene coding for the adapter protein ASC) (Nuvolone et al., 2015). Further studies are required to clarify the specificity of inflammasome activity in response to prion activation.

Furthermore, a notable characteristic of neurodegenerative diseases is the prevalent high levels of proinflammatory cytokines, IL-1β, IL-16 and TNF-α (Heneka et al., 2014). It is considered that these substances have local origins and are mainly produced by microglial cells. Because these diseases are sterile-type damage, the main DAMPs involved in neurodegeneration are fibrillar and soluble β-amyloid, α-synuclein (α-syn), ATP, LPC and the high-mobility group box protein 1 (HMGB1) (Labzin et al., 2018).

**FIGURE 3:**
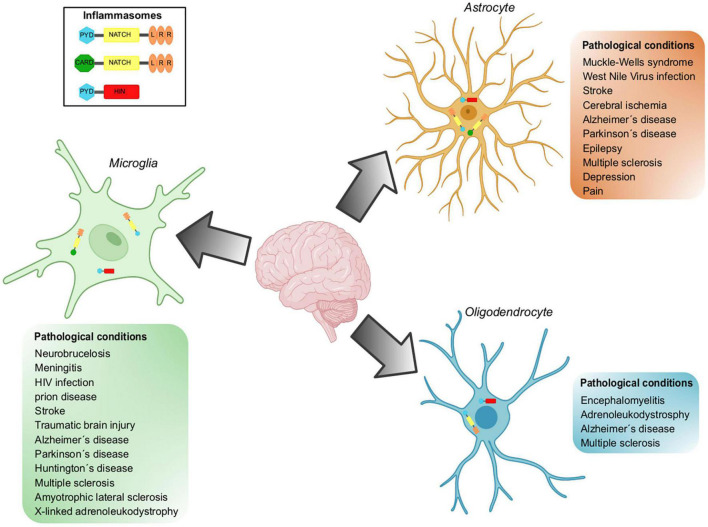
Inflammasomes implicated in pathological conditions in glial cells. Inflammasomes can be activated in the CNS in response to different stressors. Inflammasome activation has been demonstrated in resident cell types of the CNS, including microglia, astrocytes, and oligodendrocytes. NLRP3, NLRCs and AIM are the inflammasomes located in glial cells and whose participation has been demonstrated in a wide variety of pathological conditions.

There is evidence showing that microglial cells play a role in neurodegenerative diseases associated with neuroinflammation (Figure 3). In the following lines, we will describe the main results supporting this statement.

### Alzheimer’s Disease

Alzheimer’s disease is a highly prevalent form of dementia characterized by the accumulation of extracellular amyloid beta (Aβ) plaques in the brain, insoluble filaments of tau —the microtubule-associated protein accumulated as neurofibrillary tangles in the brain— neuronal cell death, and neuroinflammation. It has been demonstrated that the concentration of IL-1β and IL-18 is increased in this disease as a consequence of NLRP3 inflammasome activation (Medeiros et al., 2011; Heneka et al., 2013; Saresella et al., 2016b; Awad et al., 2017; La Rosa et al., 2019).

The CNS resident microglia population (Sarlus and Heneka, 2017; Shi and Holtzman, 2018) primarily drives neuroinflammation in AD. Although it is recognized that microglia may exert benign and reparative activities in AD through the phagocytic removal of Aβ deposits, the accumulation of Aβ may also prime microglial cells and promote their activation to produce inflammatory mediators. Moreover, upon Aβ accumulation, microglial cells may become progressively impaired in their ability to phagocytize Aβ plaques (Sarlus and Heneka, 2017; Shi and Holtzman, 2018).

Besides the elevated expression of IL-1β, the presence of Aβ plaques in microglia of AD patients has been reported (Griffin et al., 1989; Simard et al., 2006). On the other hand, *in vitro* studies have shown that fibrillar Aβ activates the NLRP3 inflammasome when phagocytized by microglia, leading to the activation of caspase-1 and the release of IL-1β (Halle et al., 2008).

Furthermore, indirect inhibition of NLRP3 inflammasome activation by clinically approved fenamate, as well as non-steroidal anti-inflammatory drugs that target cyclooxygenase enzymes and volume-regulated anion channels (VRAC), suppressed microglia-mediated neuroinflammation and memory loss in 3 × TgAD mice (Daniels et al., 2016). Collectively, these findings suggest that misfolded Aβ activates the microglial NLRP3 inflammasome, which triggers the release of pro-inflammatory factors that perpetrate a chronic neuroinflammatory environment and promote AD pathology.

Although the focus has been on the function of NLRP3 in AD, the role of other inflammasomes has also been characterized in the context of AD. It has been reported that AIM2 inflammasome increases Aβ deposition, microglia activation, and cytokine production, but it does not affect behavior or memory in transgenic 5xFAD mice (Wu et al., 2017).

### Multiple Sclerosis

This pathology is an autoimmune demyelinating disease of the CNS characterized by immune cell infiltration from the periphery into the CNS as well as by the activation of the microglia and astrocytes, which together promote neuroinflammation and neurodegeneration (Baecher-Allan et al., 2018). Most studies on the involvement of inflammasomes in multiple sclerosis (MS) have focused on the peripheral immune response that is shaped by lymphocytes and macrophages that enter the CNS during this pathology. However, direct genetic evidence points to the relevance of inflammasome signaling in microglia and border-associated macrophages during experimental autoimmune encephalomyelitis (EAE), a widely used rodent model of MS (Ransohoff, 2012; Voet et al., 2018). Also, it has been shown that the anti-inflammatory protein A20 negatively regulates NLRP3 inflammasome activation (Vande Walle et al., 2014), and the deletion of A20 in microglia and CNS macrophages exacerbates EAE in mice due to NLRP3 hyperactivation, resulting in increased IL-1β secretion and CNS inflammation (Voet et al., 2018).

CNS-intrinsic inflammasome activation was further reported in another study that showed caspase-1 and GsdmD mediated pyroptosis in microglia, as well as in myelin-forming oligodendrocytes (ODCs) in the CNS of MS patients and EAE mice (McKenzie et al., 2018).

Furthermore, the elevated expression of P2X7 receptor, a purinergic receptor that detects and amplifies the release of ATP and, therefore, the activation of NLRP3 inflammasome, was shown in spinal cords of MS patients (Yiangou et al., 2006; Matute, 2007). Taken together, these results seem to suggest that endogenous metabolic danger signals, ATP, and uric acid are likely involved in the activation of the NLRP3 inflammasome pathway observed in MS.

### Parkinson’s Disease

Parkinson’s disease is a progressive neurodegenerative disorder mostly characterized by the depletion of dopaminergic neurons in the substantia nigra and the accumulation of cytoplasmic inclusions of fibrillar α-syn, also called Lewy bodies (Petrucci et al., 2014). Different intracellular mechanisms allow the release of α-syn outside of the cell (Lee, 2008), but the common endpoint of α-syn accumulation is the activation of astrocytes and microglia to produce IL-1β (Lee, 2008; Béraud and Maguire-Zeiss, 2012). Notably, this phenomenon also facilitates the recruitment of immune cells from the periphery into the CNS (Harms et al., 2017).

Most of the evidence linking PD to inflammasome signaling comes from *in vitro* studies, but the importance of inflammasomes for the disease is not completely understood. α-Syn was shown to trigger activation of the NLRP3 inflammasome in human monocytes and BV2 microglial cells (Codolo et al., 2013; Gustot et al., 2015; Zhou et al., 2016), but not in primary microglia (Gustin et al., 2015).

Additionally, mutations in Parkin, PARK2, PARK6, and PINK1 have been identified in patients with autosomal recessive early-onset PD, and microglia and macrophages from PARK2 and PINK1 knockout mice and patients with PARK2 mutations have been shown to display an exacerbated NLRP3 inflammasome response, possibly due to impaired expression of the anti-inflammatory protein A20 that negatively regulates NLRP3 inflammasome activation (Mouton-Liger et al., 2018).

Although several studies have shown that α-syn can elicit the activation of inflammasomes in monocyte and microglial cell lines and in PD animal models, the possible role of NLRP3 in patients with a diagnosis of PD still needs to be clarified.

### Amyotrophic Lateral Sclerosis

Amyotrophic Lateral Sclerosis (ALS) is a neurodegenerative disease characterized by the selective loss of motor neurons in the motor cortex, the brainstem, and the spinal cord, which leads to muscle weakness and wasting (Taylor et al., 2016).

Neuroinflammation has an important role in the pathogenesis of ALS, as demonstrated by lymphocyte and macrophage infiltration in the CNS, microglial activation, and the presence of reactive astrocytes in the same anatomical sites where motor neuron injuries are observed. Some studies have suggested that deregulated and excessive inflammasome activation contributes to the neuroinflammation observed in this disease (McCombe and Henderson, 2011; Lall and Baloh, 2017).

Furthermore, data obtained from G93A-*SOD1* transgenic mice, the most common animal model for ALS, showed the activation of caspase-1 and IL-1β in the microglia by ALS-linked mutant SOD1 and demonstrated that caspase-1 or IL-1β gene knockout or the use of recombinant IL-1Ra resulted in a reduction of inflammation. Notably, augmented caspase-1 and IL-1β production appeared to be NLRP3-independent in this model, suggesting the possible involvement of other inflammasome complexes (Meissner et al., 2010; Lehmann et al., 2018). However, other analyses performed in the *SOD1* transgenic mice showed an upregulation of NLRP3 and ASC in the anterior dorsal thalamic nucleus of G93A mice (Debye et al., 2018) and the transactive response DNA-binding protein-43 (TDP-43) in the microglia (Zhao et al., 2015). Additionally, the authors noticed the microglial expression of ASC but not that of NLRP3, suggesting that other inflammasome sensor molecules may play a role in microglia-driven neuroinflammation in ALS.

On the other hand, clinical studies using an IL-1 receptor antagonist in ALS patients have not shown a significant reduction in disease progression (Maier et al., 2015), suggesting that inflammasome activation does not play a major role in ALS, or that the pathology is driven by IL-18 or by DAMPs *via* pyroptosis.

### Huntington’s Disease

Huntington’s Disease (HD) is an autosomal dominant progressive neurodegenerative disease caused by the expansion of a trinucleotide CAG repeat in the 5′ coding region of the Huntingtin gene, leading to the expression of an abnormal protein that gradually damages cells in the brain (Caron et al., 2018). Caspase-1 activation can be detected in the brains of HD patients and in mouse models of HD, whereas caspase-1 inhibition was shown to slow down disease progression in the R6/2 mouse model of HD (Ona et al., 1999). Mechanistically, caspase-1 was shown to cleave mutant and wild-type Huntingtin *in vitro* (Wellington et al., 1998) and *in vivo* (Ona et al., 1999), potentially contributing to the neurodegeneration seen in HD. Similarly, treatment with the tetracycline derivative minocycline delayed disease progression by inhibition of caspase-1 and caspase-3 expression (Chen et al., 2000).

### X-Linked Adrenoleukodystrophy

This genetic disease that affects the nervous system and the adrenal cortex is caused by a loss-of-function mutation of the gene encoding for the ATP-binding cassette subfamily D member 1 (ABCD1). ABCD1 is involved in the transport of very-long-chain fatty acids from the cytosol to peroxisomes, a critical step for their oxidation and disposal. In the brain, dysfunctional ABCD1 causes axonopathy of the spinal cord and inflammatory demyelination (Kemp et al., 2016). 25-Hydrocholesterol (25-HC) is a downstream product of cholesterol 25-hydroxylase, and this enzyme is increased in X-linked adrenoleukodystrophy (X-ALD) patient-derived induced pluripotent stem cells. Indeed, 25-HC activates microglia and increases cerebral IL-1β levels when injected into the murine corpus callosum (Jang et al., 2016). In LPS-primed mouse microglia, 25-HC causes NLRP3- dependent caspase-1 cleavage, leading to the release of IL-1β. This activation also requires 25-HC-induced K^+^ efflux and mitochondrial ROS production. Additionally, NLRP3 deficiency or treatment with IL-1 receptor antagonist protein (IL-1ra) decreased microglial activation upon cerebral 25-HC injection and prevented ODC cell death (Jang et al., 2016).

### Other Maladies

Neuroinflammation plays a crucial pathological role in stroke, and IL-1β has been identified as a key cytokine in this pathology. So far, four distinct inflammasomes have been implicated in stroke: NLRP1, NLRP3, NLRC4, and AIM2 (Barrington et al., 2017). NLRC4- and AIM2-deficient mice were shown to have significantly smaller infarct volumes compared to wild-type mice subjected to middle cerebral artery occlusion, which was associated with strongly reduced microglia cell activation and leukocyte recruitment to the infarct site (Denes et al., 2015).

In patients with traumatic brain injury (TBI), higher levels of inflammasome markers in their cerebrospinal fluid have been detected, including ASC, caspase-1, NLRP1, and NLRP3 (Adamczak et al., 2012; Wallisch et al., 2017). Nevertheless, NLRPβ and ASC knockout mice did not show any improvement in motor recovery or lesion volume in conditions of TBI, compared to control mice, although their levels of IL-1β were reduced (Brickler et al., 2016). NLRP3 expression localizing to neurons, microglia, and astrocytes was also detected in experimental TBI (Liu et al., 2013), and NLRP3-deficient mice as well as pharmacological blockade of NLRP3 activation were shown to improve recovery from TBI (Irrera et al., 2017); demonstrating that inflammasome activation plays an important role in this pathological condition.

Regarding spinal cord injury (SCI), it has been demonstrated that this condition induced higher levels of NLRP1, ASC, caspase-1, IL-1β, and IL-18 in mice, and therapeutic neutralization of ASC was proposed to result in tissue sparing and functional improvement due to reduced inflammasome activation in neurons (de Rivero Vaccari et al., 2008). Besides, in SCI, enhanced NLRP3 expression has been demonstrated predominantly in neurons, but also in microglia and astrocytes (Zendedel et al., 2016).

Given that microglial cells are essential for the regulation of inflammasome activities in the brain, further research into the regulation and control of these responses is required for treating diverse pathologies and developing a deep understanding of cellular damage in the brain.

## Inflammasomes and Astrocytes

Some years ago, the expression and aggregation of inflammasomes in astrocytes was a controversial topic, since it had been reported that inflammasomes were exclusively expressed in microglial cells (Gustin et al., 2015). However, more contemporaneous and overwhelming experimental evidence supports that astrocytes also express functional inflammasomes. A search in PubMed with the terms “inflammasome” and “astrocyte” resulted in 260 references (March 2022); 84% of these references (219 reports) have been published in the last 4 years, since 2018.

It is now clear that astrocytes have the capacity to assemble different types of inflammasomes and play relevant pro-inflammatory roles in neurological diseases such as depression, epilepsy, PD, AD, pain, ischemic damage, and others (Nourbakhsh et al., 2021). In a complementary context, some examples of how isolated astrocytes are able to react to known DAMPs, such as HMGB1 and ATP, are the reports by Minkiewicz et al. (2013), Yao et al. (2019), and Wang et al. (2020). In the first, HMGB1 promoted NLRP3 inflammasome formation *via* NF-κB nuclear translocation in a signaling pathway sensitive to PPARγ activation; in the second, the LPS-induced inflammation response was dependent on endoplasmic reticulum stress activation; in the third, extracellular ATP induced the aggregation of NLRP2 through the activation of purinergic P2X7 receptor; this effect was sensible to the specific antagonist reactive blue. In addition, ATP actions required the association of P2X7 receptor with Pannexin-1, as revealed by the inhibitory action of probenecid over pre-incubated astrocytes, which blocked the ATP-dependent activation of pro-caspase-1. Interestingly, silencing NLRP2 expression reduced the innate immune response mediated by ATP.

Isolated astrocytes are also able to react to the presence of ethanol, a compound that generates a pro-inflammatory state associated with an intense upregulation of ROCK2 (a serine-threonine kinase), and enhanced oxidative stress (Li et al., 2020).

It was also demonstrated in mice sensitized with LPS and primed with LPC that both canonical and non-canonical inflammasomes, namely NLRP3 and NLRC4, are assembled in microglia and astrocytes, and in consequence produce microgliosis and astrogliosis (Freeman et al., 2017). NLRC4 was initially characterized by its interaction with pro-caspase-1 through the CARD domain present in both proteins (Geddes et al., 2001; Poyet et al., 2001). In astrocytes, the activation of NLRC4 involved ASC, caspase-1, Ca^2+^ mobilization and K^+^ efflux (Freeman et al., 2017).

Numerous reports have demonstrated an outstanding role of the inflammatory process within astrocytes in different experimental models of neurological maladies (Figure 3) as well as neurological damage related to pharmacological treatments.

### Muckle-Wells Syndrome

It has also been shown that human astrocytes have the capacity to express NLRP2, a cytosolic inflammasome that is associated with ASC and caspase-1, inhibiting the activation of the transcriptional factor NF-κB (Bruey et al., 2004). A variant of this protein complex has been related to the Muckle–Wells syndrome (Fontalba et al., 2007), a rare genetic disease characterized by pro-inflammatory episodes of fever and rashes.

### Multiple Sclerosis

Multiple sclerosis is a chronic disease characterized by demyelination and axonal damage in the nervous system. It is accompanied with severe cognitive deficits and eventually becomes a disabling condition. Using the model of autoimmune encephalomyelitis, an animal model of multiple sclerosis, Hou et al. (2020) reported the generalized activation of the NLRP3 inflammasome that was associated with the alteration of the astrocyte phenotype. This group demonstrated that the experimental MS converted astrocytes to the neurotoxic phenotype A1, an action that was prevented by an IL-18 antagonist, which is related to the NLRP3 effect *via* the NF-κB pathway.

### Alzheimer’s Disease

Neurodegeneration associated with diverse forms of AD, including transgenic experimental models, is the subject of intense analytical scrutiny given the pressing health issue that this illness has become for modern societies. It is well accepted that neuroinflammation is one of the pathological events associated with AD, and that astrocytes are one cellular type with the capacity to participate in this phenomenon (Bandyopadhyay, 2021). In this context, efforts have been made to ameliorate neuroinflammation in AD to find a therapeutic strategy that improves the outcome of the patients. For example, it has been proposed that glucagon-like peptide-1 analogs could mitigate astrocytic NLRP2 activation in 5xFAD transgenic mice, improving cognitive dysfunction *in vivo* and protecting astrocytes *in vitro* (Zhang et al., 2022a); in APP/PS1 mice, AVE0991 —an angiotensin-(1-7) analog— was effective in inhibiting astrocyte-mediated neuroinflammation *via* the SNHG14/miR-223-3p/NLRP3 pathway (Duan et al., 2021). Treatment with Aβ to astrocytes promotes NLRP3 inflammasome activation. Ebrahimi et al. (2018) and Hong et al. (2019) reported neuroprotective actions of α1-antitrypsin and progesterone, mitigating the inflammatory process by downregulating the expression of NLRP3 in the first study and enhancing the autophagy-lysosomal pathway in the second.

### Parkinson’s Disease

Parkinson’s disease is now recognized as a neurodegenerative entity that involves much more than a dopamine deficit. In this context, it has also been postulated that astrocytic inflammation plays a role in the onset of this pathology (Miyazaki and Asanuma, 2020). In the experimental model of PD by MPTP treatment, it was shown in mice that the neuroinflammation process was associated with upregulation of the NLRP3 inflammasome and glia maturation factor. This factor was coincident with the presence of α-syn in an astrocyte population. The authors demonstrated that molecular ablation of the glia maturation factor significantly reduced the presence of NLRP3 in astrocytes (Javed et al., 2020). Again, with mice treated with MPTO, Zhu et al. (2018) reported a protective role for dopamine D2 receptor agonist inhibiting astrocytic NLRP3 inflammasome activation *via*β-arrestin 2. It was shown in primary mouse astrocytes and in the U373 cell line that the tumor necrosis factor-like weak inducer of apoptosis (TWEAK) treatment enhanced the activation of the Stat3/NLRC4 inflammasome signaling axis with the participation of PKCδ. TWEAK has been involved in PD-like neuropathology (Samidurai et al., 2020). Given that Parkinsonian patients experience olfactory deficits, Zhang et al. (2020a) treated mice with rotenone to simulate olfactory disturbances in PD. These authors reported a role of the mitochondrial fission factor Drp1 in the promotion of pro-oxidant responses that favored NF-κB nuclear translocation and subsequent NLRP3 inflammasome activation.

### Viral Infections

Astrocytes also express AIM2 inflammasome, and its assembly is efficiently induced by double-stranded DNA, suggesting a role of AIM2 in detecting DNA viruses. In response to mimetic DNA, AIM2 activation induced the synthesis of IL-6 and INF-γ (Cox et al., 2015). It was reported by Soung et al. (2022), that after an infection by West Nile Virus, the recovery of the resultant encephalitis was associated with continued astrocyte inflammasome-mediated production of IL-1β, which was maintained by hippocampal astrogenesis *via* IL-1R1 signaling in neural stem cells.

### Epilepsy

Seizures and epilepsy are another neuropathology that has been related to inflammatory responses. In mice treated with kainic acid, they presented an epileptic outcome that was associated with NLRP3 activation; the inflammasome promoted an epileptic crisis by enhancing the expression of astrocytic adenosine kinase. The mechanism of this action was further explored using astrocytes in culture, and it was demonstrated that adenosine kinase upregulation depended on the CREB/REST/SP1 signaling pathway (Zhang et al., 2022b).

### Cerebral Ischemia and Stroke

Both NLRP2 and NLRP3 have been shown to participate in the proinflammatory events related to experimentally induced cerebral ischemia after cerebral ischemia-reperfusion injury and acrolein-induced astrocytic inflammation in brain affected by ischemic stroke. Cheon et al. (2018) demonstrated that cerebral ischemia activated NLRP2 inflammasome in a set of events that were dependent on the apoptosis signal-regulating kinase 1 (ASK1), a finding that was confirmed in an astrocyte cell line that was subjected to oxygen-glucose deprivation and reperfusion injury. A study using the model of middle cerebral artery occlusion as well as TNA2 astrocytes (astrocytic cell line containing the oncogenic early region of SV40) exposed to oxygen-glucose deprivation showed that gastrodin (GAS) was able to inhibit NLRP3 and NLRC4 expression (Sui et al., 2019). Liu et al. (2020) reported the suppression of NLRP3 inflammasome in astrocyte treatment with adiponectin in a model of cerebral ischemia-reperfusion injury. The protective adiponectin action was mediated by the AMPK/GSK-3β signaling pathway. In another report, it was shown that acrolein, a neurotoxin produced by pro-oxidant reactions in tissues affected by ischemic stroke, promoted NF-κB and NLRP3 inflammasome activation. This action was mediated by the induction of ADAM10 and the participation of p38 MAPK (Park et al., 2020).

### Pain

It has been reported that pain-related clinical situations and experimental models are associated with inflammatory response activation in astrocytes (Tan et al., 2021). Both NLRP2 and NLRP3 have been identified in spinal cord astrocytes during in situations of mechanical pain hypersensitivity in the complete Freund adjuvant-induced persistent pain model with IL-1β as effector and by activating the sphingosine-1-phosphate receptor (Doyle et al., 2019; Ducza et al., 2021). It has been shown that the inflammatory activation in spinal cord astrocytes, and the associated pain, can be alleviated by treatment with the stilbenoid resveratrol (Fan et al., 2021), and the molecular reduction of heat shock protein family A member 8 (HSPA8) (Mi et al., 2021). In a different model, Carranza-Aguilar et al. (2022), reported that daily treatment with morphine and fentanyl differentially induced cell-specific activation of NLRP3 inflammasome and pyroptosis in the dorsal raphe nucleus through TLR4 receptors in astrocytes and opioid receptors in neurons, indicating that neuroinflammation is involved in opioid-induced analgesia and fentanyl-induced hyperalgesia.

### Depression

Depression is one of the most complex mood disorders. It is usually a long-term disabling condition that has an enormous repercussion in the health system. In both clinical and experimental model observations, it has been recognized that inflammatory events play an important role in this neuropathological condition, including alterations presented by astrocytes (Kim et al., 2018). By using sleep deprivation as an inducer of the depressive condition, one study showed that NLRP3 activation in astrocytes reduced BDNF levels, whereas combined treatment with fluoxetine and leptin ameliorated the depression induced by sleep deprivation (Li et al., 2019). Depressive mice were shown to present astrocytic loss that was promoted by pyroptotic cellular death. The pyroptotic process was triggered by the activation of the NLRP3/caspase-1/GSDMD pathway (Li et al., 2021). Also, in mice with depression induced by chronic mild stress, it was reported that kynurenine, a tryptophan metabolite, acted as a pro-inflammatory factor in astrocytes. In this model, hippocampal astrocytes expressed NLRP2 inflammasome *via* NF-κB nuclear translocation (Zhang et al., 2020b). In mice with molecular ablation of neuroligin3, it was shown that this factor is needed for NLRP3 inflammasome activation in a chronic unpredictable mild stress model. Hence, suppression of neuroligin3 down-regulated NLRP3 and the ASC protein, preventing the loss of astrocytic cells during depressive episodes (Li et al., 2018).

### Astrocytic Inflammation in Non-pathological Conditions

Inflammasomes in astrocytes are relevant not only in pathological entities, but also as part of several physiological and protective responses. For example, NLRP3 deficiency in mice is associated with hippocampal dysfunction as well as anxiety-like behavior (Komleva et al., 2021). Absence of NLRP3 renders astrocytes highly permissive to *Trypanosoma cruzi* replication in the context of Chagas disease (Pacheco et al., 2019). Mice treated with LPS in their hippocampus responded by enhancing the astrocytic production of neopterin, a biomarker for immune system activation; neopterin inhibited inflammasome activation in pre-conditioned human astrocytes (de Paula Martins et al., 2018).

Although microglia were once considered the only cell type related to immunity in the brain, it is now clear that inflammasome aggregation also occurs in astrocytes. This fact reveals a new paradigm in the immune responses of the CNS with implications for the functional concepts of cell regulation in the brain and for the development of new therapeutic strategies against neurodegenerative diseases.

## Inflammasomes and Oligodendrocytes

Oligodendrocytes are specialized glial cells in the CNS whose main tasks include the production of myelin sheath to wrap and electrically insulate neuronal axons, provide metabolic support to axons and participate in neuroplasticity processes; their lineage in the CNS includes immature precursors (OPCs) and mature ODCs (Baumann and Pham-Dinh, 2001; Zhou et al., 2021).

Seminal work suggested a contribution of inflammasome-mediated responses to demyelinating pathologies whose main target are ODCs. Thus, in a demyelination model induced by cuprizone feeding in mice, the expression level of *Nlrp3* transcript incremented dramatically (> 100 fold); in agreement, in *Nlrp3* knockout mice, cuprizone-induced inflammation, demyelination and ODC death were significantly delayed (Jha et al., 2010), suggesting localized activity of Nlrp3 inflammasome in ODCs. In EAE, an animal model of MS, a role was also demonstrated for the adaptor protein PYCARD/ASC and caspase-1, but the sensor protein remains elusive (Shaw et al., 2010).

On the other hand, in childhood cerebral adrenoleukodystrophy (CC-ALD), the most severe form of X-ALD, characterized by severe demyelination, upregulation of *CH25H* transcript (coding for cholesterol 25-hydroxilase) was found. The product of this enzymatic activity, 25-hydroxycholesterol (25-HC), resulted a potent inductor of neuroinflammation in CC-ALD. CH25H mRNA was specifically incremented in OPCs differentiated from pluripotent stem cells (iPSCs) isolated from CC-ALD patients. In agreement, injection of 25-HC into the corpus callosum induced microglia recruitment and IL-β production, which conduced to apoptotic ODC death. These effects were significantly attenuated in Nlrp3-deficient mice, suggesting an inflammatory response dependent on NLRP3 inflammasome in ODCs (Jang et al., 2016).

Distribution of NLRP1/NALP1 and NLRP3/NALP3 in several tissues of the body was explored using self-designed monoclonal antibodies, and it was detected that NLRP1 inflammasome is expressed in ODCs (Kummer et al., 2007). Despite these approaches, inflammasome activity in ODCs was only recently recognized.

In a study aimed at investigating if inflammasomes are activated in MS, a neurological disease characterized by loss of neuron-covering myelin, it was observed that in homogenates from postmortem brain samples of MS patients, transcripts from I-1β, IL-18, CASP1, GsdmD, NLRP3, NLRP1 and AIM2 incremented in comparison with control samples; in the same biopsies, it was documented by immunofluorescence that GsdmD co-localized with glutathione S-transferase-π^+^ (GST-π^+^), a marker of mature ODCs, indicating specific inflammasome activity in these glial cells (McKenzie et al., 2018). Furthermore, in mature ODCs *in vitro*, it was shown that in response to a challenge with TNF-α, the expression level of CASP1 and GsdmD transcripts and their respective proteins incremented. These changes were concomitant with cell death-related morphological changes, such as a condensed nucleus and short and few cellular processes in a caspase-1 dependent way, indicating the induction of pyroptosis (McKenzie et al., 2018).

Demyelination is one of the main alterations of the white matter observed in AD (Desai et al., 2009; Zhan et al., 2014). Studies on mature ODCs from AD patients and AD transgenic mice demonstrated that NLRP3 inflammasome is active in ODCs and participates in pathologic demyelination through a pathway triggered by hyperactivated Drp1, a mitochondrial GTPase involved in organelle division (Smirnova et al., 2001); this factor inhibits hexokinase 1 (HK1), which in turn activates NLRP3 inflammasome. This metabolic stress and chronic inflammation conduce to demyelination and tissular degeneration of white matter and contribute to the cognitive impairment of AD (Zhang et al., 2020c).

The exposed evidence supports the notion that deleterious neuroinflammation observed in pathologies where demyelination is characteristic, is substantially supported by inflammasome activity (Figure 3).

## Concluding Remarks

Inflammasomes are multiprotein machineries mediating maturation and secretion of IL-β and IL-18 in response to various sterile and infectious stimuli. Inflammasomes are functional in microglial cells and, surprisingly, in astrocytes and ODCs as well, and they mediate responses to specific stressors. However, chronic activation or mutations in inflammasomes lead to a variety of diseases (Figure 3). Obtaining detailed information about affected cell populations, molecular identity and function is an important opportunity for the development of innovative therapies.

## Author Contributions

All authors listed have made a substantial, direct, and intellectual contribution to the work, and approved it for publication.

## Conflict of Interest

The authors declare that the research was conducted in the absence of any commercial or financial relationships that could be construed as a potential conflict of interest.

## Publisher’s Note

All claims expressed in this article are solely those of the authors and do not necessarily represent those of their affiliated organizations, or those of the publisher, the editors and the reviewers. Any product that may be evaluated in this article, or claim that may be made by its manufacturer, is not guaranteed or endorsed by the publisher.
